# WHO preferred product characteristics for bivalent Salmonella Typhi/Paratyphi A vaccine for comprehensive protection against enteric fever– key considerations and research gaps

**DOI:** 10.12688/gatesopenres.16364.1

**Published:** 2025-09-15

**Authors:** Ana Belen Ibarz Pavon, John Clemens, Alejandro Craviotto, John A. Crump, Denise O. Garrett, Melita A. Gordon, Jacob John, Karen H. Keddy, Matthew B. Laurens, Xinxue Liu, Florian Marks, Andrew J. Pollard, Senjuti Saha, Annelies Wilder-Smith

**Affiliations:** 1 World Health Organization. Department of Immunization, Vaccines and Biologicals, Geneva, Switzerland; 2 International Vaccine Institute, Seoul, South Korea; 3 UCLA Fielding School of Public Health, Los Angeles, USA; 4 Korea University School of Medicine, Seoul, South Korea; 5 Universidad Autonoma de Mexico. Facultad de Medicina, Mexico City, Mexico; 6 University of Otago. Centre for International Health., Dunedin, New Zealand; 7 Sabin Vaccine Institute, Washington, District of Columbia, USA; 8 University of Liverpool. Department of Infection, Veterinary & Ecological Sciences, Liverpool, UK; 9 Malawi-Liverpool Wellcome Trust Program. Kamuzu University of Health Sciences, Blantyre, Malawi; 10 Christian Medical College Vellore, Vellore, Tamil Nadu, India; 11 University of Pretoria. Faculty of Veterinary Science. Department Veterinary Tropical Diseases, Pretoria, South Africa; 12 University of Maryland Center for Vaccine Development and Global Health, Baltimore, Maryland, USA; 13 University of Oxford Oxford Vaccine Group, Oxford, England, UK; 14 Child Health Research Foundation, Dhaka, Dhaka Division, Bangladesh

**Keywords:** Salmonella enterica, typhoid fever, paratyphoid fever, vaccine

## Abstract

In 2021, *Salmonella* Paratyphi A caused >2 million illnesses, resulting in >14,000 deaths, most of which occurred among children under 5 years of age in socioeconomically deprived populations. Both typhoid fever and paratyphoid fever occur in such areas, but paratyphoid fever is currently concentrated in South Asia. Typhoid conjugate vaccines are recommended for the control of enteric fever in typhoid-endemic settings; however, there are increasing demands for the development of vaccines that can address enteric fever more broadly by including protection against paratyphoid fever. The WHO preferred product characteristics (PPC) and a research and development (R&D) technology roadmap are normative documents developed with the guidance and contribution of a multidisciplinary expert group following a standard methodological framework. In this paper, we summarize the PPC and R&D roadmap presenting the key attributes for a bivalent *Salmonella enterica* serovar Typhi and Paratyphi A vaccine, and discuss the identified key research and data gaps needed to optimize vaccine value and to inform public health and policy decisions, with a particular focus in paratyphoid and enteric fever endemic countries.

## Introduction

*Salmonella enterica* serovar Typhi and *Salmonella enterica* serovar Paratyphi A cause typhoid and paratyphoid fever respectively, which are collectively referred to as enteric fever. Paratyphoid fever may be caused by *Salmonella* serovars Paratyphi A, B or C, with *S.* Paratyphi A currently predominating. ^1^ *Salmonella* serovars Typhi and Paratyphi A are restricted to human hosts, and transmission occurs through the faecal-oral route *via* the ingestion of faecally-contaminated food and water. ^2^^,^^3^ The disease incubation period is typically 7 to 14 days, and commonly presents with fever, malaise, and mild gastrointestinal symptoms, often indistinguishable from other febrile illnesses. ^3^^,^^4^

Globally, in 2017 there were an estimated 11-27 million cases of enteric fever, and >120,000 associated deaths; 75% of illnesses and >85% of deaths are caused by *Salmonella* Typhi. ^5^ There is, however, evidence that *Salmonella* Paratyphi A is responsible for a growing proportion of enteric fever, and in endemic countries such as India or Nepal, paratyphoid fever can account for >40% of enteric fever. ^6^^–^^8^ Moreover, the unavailability of reliable routine diagnostic capacity and weak surveillance systems likely result in an underestimation of the true contribution of *S.* Paratyphi A to the burden of enteric fever. ^9^^,^^10^

Geographically, typhoid fever is found worldwide and presents a major public health problem to countries in Asia and sub-Saharan Africa, whereas paratyphoid fever is concentrated in, but is not limited to, countries in Asia and the Middle East. Both typhoid and paratyphoid fever are associated with unsanitary living conditions and lack of access to microbiologically safe water and food. While high population density in urban slums is a known risk factor for both typhoid and paratyphoid fever, typhoid fever in Africa is also frequently found in rural, low-population density sites across the continent. ^11^^,^^12^ There are substantial variations on the age distribution of both typhoid and paratyphoid fever across geographies and over time; however, enteric fever appears to be common among young children, and a substantial burden of disease remains until early adulthood. While the highest typhoid fever incidence rates are often found among children under five years of age, paratyphoid fever appears to peak later in life, often between 5 and 15 years of age. ^5^

Outcomes for enteric fever are improved with timely and appropriate antimicrobial therapy. However, antimicrobial resistance (AMR) is a growing concern. Resistance to traditional first-line treatment - ampicillin, chloramphenicol, and cotrimoxazole - among typhoidal *Salmonella* strains has been reported since the 1950s. ^13^ Resistance to all three antimicrobials, or multidrug resistance (MDR), has been documented since the 1980s. ^14^^,^^15^ The subsequent use of nalidixic acid and ciprofloxacin as alternative treatments for resistant enteric fever led to the emergence of fluoroquinolone non-susceptibility (FQNS) and ciprofloxacin resistance. Extensively drug resistant (XDR) strains are resistant to traditional first-line antimicrobials, fluoroquinolones, and third-generation cephalosporines: these emerged in Pakistan in 2016 and have spread worldwide through international travel. ^16^^–^^19^ While antimicrobial resistance patterns including MDR vary by place and serotype, both *S.* Typhi and *S.* Paratyphi A are currently reported to present FQNS at a prevalence of >90%, while XDR strains have been identified only in *S. serovar* Typhi to date.

Access to microbiologically safer water and food, sanitation, and hygiene (WASH) practices remains important measures for the prevention of enteric fever. ^21^ However, in the absence of the resources required for the provision and development of such infrastructures, vaccines are a more viable alternative. Safe and effective vaccines against typhoid fever exist, and their programmatic use has been recommended by the World Health Organization (WHO) since 2008. The most recent WHO position paper, updated in 2018, recommends the use of typhoid conjugate vaccines (TCV) in the infant immunization schedule from 6 months of age, and promotes TCV introduction into the routine programmatic schedule at 9 months of age or in the second year of life in endemic countries with high disease burdens or with high AMR prevalence. ^22^ Single dose TCV has demonstrated high protective efficacy in paediatric and young adult populations in endemic areas, ranging from 79-85%. ^23^^–^^28^ However, evidence suggests a progressive decline of protection over time, particularly, among those who received the vaccine before the age of 2 years, and the need for a booster dose is currently under review. ^29^^,^^30^ While TCVs have been instrumental for typhoid fever control in highly endemic countries, and have successfully contributed to the containment of outbreaks caused by MDR and XDR strains, ^18^^,^^28^ the absence of a vaccine to prevent paratyphoid fever remains a critical gap in enteric fever prevention strategies. Current vaccine development efforts are focused on bivalent conjugate vaccine candidates that combine the antigen O:2, which is present in *S.* Paratyphi A, linked to a carrier protein with an existing TCV construct. ^31^^,^^32^ Some of these products are soon to start efficacy evaluations, which should corroborate existing evidence that antibody levels against the O:2 antigen correlate to serum bactericidal activity. ^31^^–^^33^ In addition, a live-attenuated vaccine candidate using the CVD 1902 strain of *S.* Paratyphi A was proven immunogenic in a phase 1 study, and it is currently being investigated for efficacy using a controlled human infection model (CHIM). ^34^^,^^35^

Vaccine developers and manufacturers rely on WHO guidance for research and development, regulation and prequalification pathways for novel vaccines. Currently, no such guidance has been released for paratyphoid vaccines, and immune correlates of protection for a paratyphoid vaccine are yet to be established. It is widely acknowledged that phase 3 efficacy trials for a *Salmonella* Paratyphi A-containing vaccines are unlikely to be logistically and economically feasible due to the lower prevalence of paratyphoid fever in comparison to typhoid fever. Hence, the WHO’s Product Development for Vaccines Advisory Committee (PDVAC) has endorsed a regulatory pathway for bivalent conjugate vaccines that would rely on data obtained from a CHIM in an adult population in a non-endemic country, paired with a phase 3 safety and immunogenicity trial in a target population, and the commitment from manufacturers to conduct post marketing effectiveness studies. ^36^^,^^37^

Following a consultative process with vaccine experts, developers, manufacturers, and policy-makers, the WHO has developed a technical research and development roadmap, and defined the preferred product characteristics (PPC) for *Salmonella* Paratyphi A-containing vaccines, aiming at guiding the work of vaccine developers, manufacturers, and funding bodies in regards to vaccine development and regulatory data requirements, ensuring that critical questions are addressed in a manner to facilitate regulatory processes of national and international bodies, and support robust policy decision-making after the products are available. ^38^^–^^41^ These documents provide considerations for bivalent conjugate and live-attenuated products, and highlight the current research gaps that might hinder decision-making in regards to the use and implementation of these vaccines in both: endemic settings where paratyphoid fever constitutes currently a public health concern, and non-endemic settings where *Salmonella* Paratyphi A could be introduced and fill in the niche that TCV could open by targeting *Salmonella* Typhi.

## Rationale and methodology

Due to the lower incidence of paratyphoid fever relative to typhoid fever and its geographic distribution, a monovalent *Salmonella* Paratyphi A vaccine is unlikely to be commercially viable. ^42^^,^^43^ However, there is growing interest in addressing the public health burden of paratyphoid fever through the development of a bivalent *S.* Typhi/Paratyphi A vaccine. ^44^^–^^46^

The development of the bivalent *Salmonella* Typhi/Paratyphi A vaccines PPC and R&D roadmap responded to the need expressed by vaccine developers and manufacturers for WHO normative guidance on the characteristics of a successful vaccine that would have credence with decision-makers in settings endemic for paratyphoid fever, facilitate the regulatory and licensure procedures, and identify the research needs to ensure essential data and information gaps can be addressed during vaccine development. To this end, the WHO convened a Technical Advisory Group on *Salmonella* Vaccines (TAG-SV), a diverse group of experts with academic, vaccine development and regulatory background who advise the WHO in matters related to the research and development of *Salmonella* vaccines.

The development of the PPC and R&D roadmap followed WHO’s established procedures for the development of normative documents. An initial baseline situation analysis was conducted through a literature review to identify current *Salmonella* Paratyphi A-containing vaccines in the development pipeline, and to assess where critical data gaps that could hinder their progression to licensure, implementation, and public health decision-making were found, and how to address these in an equitable manner. The TAG-SV conducted an iterative consultative process, structured as a collaborative effort, through technical consultations, and these were supplemented with contributions from external observers from regulatory agencies and manufacturing companies when required. Both the PPC and R&D roadmap documents underwent an open, public consultation, which received input from academic experts, industry partners, and regulatory agencies, and were reviewed and endorsed by PDVAC in December 2024.

## Vaccines under development

There are currently two bivalent conjugate products in development. Both products link the *Salmonella* Paratyphi A O:2 antigen to a carrier protein, and combine it with a conjugate construct of *Salmonella* Typhi Vi-polysaccharide. The product most advanced in the development pipeline, the Serum Institute India bivalent paratyphoid A-typhoid conjugate vaccine (SII-PTCV) uses tetanus toxin (TT) as the protein carrier for the Vi-polysaccharide from *Salmonella* Typhi, and diphtheria toxoid (DT) for the O:2 antigen. The vaccine recently completed a phase 1 study in India that included 60 adults aged 18-45 years who received a single dose of either SII-PTCV, or the monovalent typhoid conjugate vaccine Typbar-TCV. Participants were followed for up to 181 days. The vaccine had a good safety profile, with mostly mild adverse events being reported and no significant differences between the intervention and comparator groups. The vaccine was immunogenic against Vi with a 97.7% and 93.3% seroconversion for anti-Vi IgG and anti-Vi IgA, respectively, in the intervention group 29 days post-vaccination, which was comparable to that observed among Typbar-TCV recipients and was sustained to day 181. The SII-PTCV vaccine was also immunogenic against O:2 with an observed increase in Serum Bactericidal Activity (SBA) titers post-vaccination and a sustained 100% seroconversion among SII-PTCV vaccine recipients but not in the Typbar-TCV control group. ^32^

A second bivalent conjugate product that uses O:2 and Vi-polysaccharide, both linked to the CRM _197_ carrier protein is currently being developed by the GSK Vaccines Institute for Global Health (GVGH). The O:2-CRM _197_ construct was shown to be immunogenic in pre-clinical studies in animal models, and reactive against a purposedly selected panel of clinical isolates. This vaccine is now progressing towards completion of phase 1 evaluation. ^31^^,^^33^

In addition to conjugate products, an oral live-attenuated bivalent vaccine containing a *Salmonella* Typhi CVD909 strain, which is the main component of a licensed oral vaccine against typhoid fever – Ty21 - and the CVD1902 strain of *Salmonella* Paratyphi A is under development by the University of Maryland, in collaboration with Bharat Biotech International and the University of Oxford. An oral live-attenuated monovalent CVD1902 vaccine was tested in healthy volunteers who ingested either a single dose of an increasing number of colony-forming units (CFUs) up to 10 ^10^ or a placebo. The study showed that a single dose of at least 10 ^9^ CFUs of CVD1902? was capable of eliciting cell-mediated immune responses that had the potential to be protective against *S.* Paratyphi A infection. ^34^ This vaccine has recently completed a CHIM, and preliminary results indicate that a CVD1902-containing oral vaccine is efficacious in protecting against *S.* Paratyphi A infection. The next step is now to combine the two strains: the typhoid CVD909 and paratyphoid CVD1902. ^35^^,^^47^

## Vaccine preferred product characteristics

Following the successful development and introduction of vaccines against typhoid fever, the logical next step is the development of a bivalent vaccine for broader control of enteric fever. Public health authorities across endemic and non-endemic countries have increasingly identified a bivalent vaccine containing a *Salmonella* Paratyphi A component as a critical need. Concern of increasing proportion of enteric fever cases caused by *S.* Paratyphi A, fear of a possible serovar replacement following the introduction of TCV, and increasing prevalence of AMR have driven demand. ^8^^,^^45^^,^^48^^–^^50^

The characteristics and use case for a bivalent *Salmonella* Typhi/Paratyphi A vaccine are mainly driven by the characteristics and most recent recommendations for TCVs. As outlined in Table 1, the expectation would be for a vaccine that can prevent both typhoid and paratyphoid fever disease and their complications. The vaccine should be suitable to be safely administered from six months of age, and be suitable for administration through the routine infant immunization scheme as well as for its use in campaigns for outbreak control and other events of public health concern, such as an increase in intestinal perforations in a context where microbiologic confirmation of the underlying cause might be constrained. The bivalent vaccine would be expected to confer protection against typhoid fever non-inferior to that observed in TCVs, and be superior to naturally acquired immunity for the *Salmonella* Paratyphi A component. Regulatory recommendations for such vaccine have already been outlined by PDVAC and WHO, ^36^^,^^37^ and post marketing evaluations will be a requirement to corroborate safety, efficacy and immunogenicity findings, and to demonstrate non-interference between the two antigens in the bivalent vaccine, and among other vaccine antigens that might be co-administered within the vaccination schedule. ^40^

**Table 1.** Preferred product characteristics for bivalent *Salmonella* Typhi/Paratyphi A vaccines. ^40^

Parameter	Preferred characteristics	Notes
**Vaccine Type**	• A **conjugated vaccine** where the *S.* Typhi (Vi polysaccharide) and *S.* Paratyphi A antigens are linked to a protein carrier. • A **live attenuated vaccine** consisting of two attenuated strains: one *S.* Typhi, and one *S.* Paratyphi A.	• A monovalent *S.* Paratyphi A vaccine is unlikely to be an attractive product, as the regions where paratyphoid fever is endemic prioritize the prevention of typhoid fever, which is currently a more prevalent aetiology.
**Target Population**	• Primarily targeted to infants from 6 months of age, to be implemented through the EPI and/or through catch-up or dedicated campaigns, and in response to specific situations such as high vulnerability or outbreaks.	• The vaccine is primarily conceived for use in routine infant immunization programs. For **conjugate products, the** recommendation for the age of vaccination will be guided by the most recent recommendations for existing TCV (currently 6 months-65 years). • Use of the vaccine in pre-implementation catch-up campaigns, and its use for the control of outbreaks and other health events will be guided by the most recent recommendations for TCV. • Special consideration to be made for infants/children who have already received at least one dose of TCV, once data on the duration of the immune response to TCV, including modelling studies data, can inform the optimal time to administer a bivalent product. • **Live-attenuated vaccines** will need to ensure the formulation is appropriate for infants and toddlers to be able to swallow the product; otherwise, an adapted age-recommendation might be required.
**Schedule**	• For a **conjugate vaccine,** a single dose schedule implemented through the routine immunization program is desirable. A two-dose schedule might be feasible. • For **live-attenuated vaccines,** it is anticipated that, for the primary schedule, multiple doses administered a few days apart will be needed.	For **conjugated vaccines, s**cheduling will be guided by the most up-to-date recommendations for TCVs. Ongoing modelling work will further inform the optimal timing for the primary dose and possible booster to ensure maximum protection during peak enteric fever age, while managing a feasible delivery strategy and cost-effectiveness.
**Safety**	• Safety and reactogenicity should be at least as favourable as existing TCV and other WHO-recommended routine parenteral and oral vaccines for use in the EPI, such as pentavalent vaccine, multivalent pneumococcal conjugate vaccine, MCV1 and MCV2, or rotavirus vaccine.	
**Efficacy Targets**	• For **conjugate vaccines,** the typhoid component of the bivalent vaccine should the efficacy target is anti-Vi immunological non-inferiority to licensed TCV. The paratyphoid component, the vaccine will be required to demonstrate superiority to natural immunity, documented as a 4-fold rise on antibody titters, or as a GMT ratio >1 compared to natural infection. • For a **live attenuated vaccine,** vaccine efficacy will need to be demonstrated to both vaccine components, as no comparator is currently available.	• While a Phase 3 efficacy trial would be the preferred standard to evaluate a *S.* Paratyphi A-containing vaccine, this has been deemed unfeasible. Alternatively, demonstration of vaccine effectiveness in a *S.* Paratyphi A CHIM could be accepted by regulators to grant licensure. • In the absence of phase 3 efficacy data post marketing phase 4 effectiveness studies will be required to corroborate vaccine efficacy.
**Serovar Coverage**	• *S.* Paratyphi A and *S.* Typhi for bivalent vaccines.	
**Adjuvant Requirement**	• No adjuvant.	• The inclusion of adjuvants might enhance reactogenicity. • Currently licensed TCV products do not contain adjuvants.
**Immunogenicity**	• For **conjugate vaccines,** the immunogenicity target of the *S.* Paratyphi A component would be superiority to naturally acquired immunity in an endemic population. • **Live-attenuated vaccines** are expected to generate an immune response targeting several antigens. There are currently no data available to identify which of these components will be relevant for immunogenicity and protection.	
**Coadministration**	• Non-immunological interference between the two vaccine components in a bivalent *S.* Typhi/Paratyphi A vaccine. • Non-immunological or safety interference when co-administered with other vaccines in the routine immunization schedule.	• Non-interference between the two vaccine antigens can be evaluated during vaccine development. • Non-interference when co-administered with other vaccines will need to be further evaluated in postmarketing phase 4 studies.
**Route of administration**	• **Conjugated vaccines** administered parenterally. Alternatively, a needle-free patch to deliver the product intramuscularly might be developed. • **Live-attenuated vaccines** administered orally as an enteric-coated capsule or a liquid formulation.	
**Registration, WHO prequalification, and programme suitability**	• Licensure by a fully- accredited national regulatory agency. • WHO prequalification following standard processes, once the vaccine meets the WHO-defined suitability criteria.	
**Vaccine Value Proposition**	• Dosage immunization regimen and cost of goods amenable to affordable supply. The vaccine should be cost-effective, and price should not be a barrier to access, including in LMICs.	• A vaccine value profile was published in October 2023. ^42^

CHIM: controlled human infection model; EPI: expanded program of immunisations; GMT: geometric mean titter; LMIC: low-and-middle income countries; LPS: lipopolysaccharide; MCV: measles-containing vaccine; TCV; typhoid conjugate vaccine.

## Research and development gaps

Table 2 outlines the research gaps identified by the TAG-SV and other experts who contributed to the development of the document. ^41^ The development and implementation of TCVs prompted substantial investment from funders, governments, and other stakeholders to address epidemiology and burden of disease data gaps in preparation to TCV introductions into routine schedules and through immunization campaigns. Laboratory and population-based regional surveillance initiatives such as the Severe Typhoid in Africa (SETA) program, the Surveillance for Enteric Fever in Asia Project (SEAP), Surveillance for Enteric Fever in India (SEFI), and the Strategic Typhoid Alliance Across Africa and Asia (STRAATA) aimed at characterising the incidence and outcomes of enteric fever, with a focus on typhoid fever. ^12^^,^^51^^,^^52^ While these surveillance initiatives have shed light into the burden and distribution of paratyphoid fever and its contribution to the overall burden of enteric fever, substantial uncertainty remains regarding the true disease incidence – particularly by age group – due to small case numbers and resulting statistical limitations. n age sub-groups. ^53^^–^^55^ Moreover, while paratyphoid fever is recognised as endemic in Asia and the Middle East, its presence in sub-Saharan Africa is increasingly being documented. ^56^^–^^59^

**Table 2.** Priority activities as expressed in the research and development technology roadmap for bivalent *Salmonella* Typhi/Paratyphi A vaccines.

Key strategic areas	Proposed priority activities
**Research**	• Implementation/improvement of enteric fever laboratory-based surveillance systems and data completeness and quality • Data reporting and burden of disease estimates • Addressing the diagnostic gaps by: ○ building and sustaining microbiology laboratory capacity for isolation, identification, and serotyping of *Salmonella* from blood culture ○ development of accurate, easy to use diagnostic alternatives to blood culture • Use of modelling to estimate the impact of a *S.* Paratyphi A-containing vaccine on disease dynamics, containment of AMR, vaccine cost-effectiveness and cost-benefit analyses
**Vaccine development**	• Define the appropriate efficacy trial design and clinical endpoints in a scenario where a phase 3 efficacy trial will not be feasible • Define correlates of protection for *S.* Paratyphi A antigens, and production of reference standards for vaccine evaluation • Clear regulatory approach and pathway to licensure at national and international level • Immune interference studies for coadministration with other vaccine antigens in the routine immunization schedule
**Public health impact**	• Clear understanding of vaccine buy-in needs for ○ Paratyphoid fever endemic countries ○ Typhoid fever endemic countries where *S.* Paratyphi A? is not currently present ○ Travellers market • Cost-effectiveness and cost-benefit analyses
**Key capacities**	• Public health workforce capacity building • Develop effectiveness and safety vigilance systems for post marketing evaluation and surveillance (including enhance laboratory capacity) • Sustainable financing mechanisms and incentives for vaccine supply • Effective communication and stakeholders' engagement

AMR: antimicrobial resistance.

Traditionally, in the absence of laboratory capacity for complete *Salmonella* identification, the term enteric fever has been equated to typhoid fever, which has resulted in an underappreciation of the role of paratyphoid fever. Furthermore, when typing does not extend to distinguishing among *Salmonella* Paratyphi A, B, and C, paratyphoid fever may be assumed to be mostly caused by *S.* Paratyphi A. ^60^^,^^61^ Building microbiology diagnostic capacity in endemic settings, and developing rapid, easy-to-use, affordable diagnostic tests that can discriminate between invasive *Salmonella* serovars remain a priority to generate good quality, complete surveillance data suitable for decision-making. Although modelling provides insight, robust primary data remain essential.

One of the main requests from vaccine developers has been for clear guidance on the regulatory processes, and the role of CHIM to evaluate vaccine efficacy in the absence of phase 3 data. The WHO’s PDVAC endorsed an alternative regulatory pathway, which would consider the evaluation of efficacy data from a CHIM in non-endemic populations, paired with an immunogenicity and safety study in no less than 3,000 subjects in an endemic, target population, and provided there is commitment from manufacturers for post marketing evaluation of safety, effectiveness, and non-interference. ^36^ WHO convened an expert consultation with academics, vaccine developers, and regulators, where all key stakeholders expressed their openness to license bivalent vaccines on those bases. ^37^

Finally, while a favourable vaccine value profile for bivalent *Salmonella* Typhi/Paratyphi A vaccines was published in 2023, ^42^ decision-making stakeholders will still need evidence of the cost-benefit and cost-effectiveness of using a bivalent vaccine, and the cost implications in comparison to the use of a monovalent TCV. For this, it is imperative that key capacities are strengthened in-country to ensure nationally-owned and operated sustainable surveillance and reporting systems for enteric fever and other invasive *Salmonella* diseases, which will, in turn, facilitate the implementation of post marketing evaluations and continuous vigilance of vaccine safety events, and generate evidence-based information to support the implementation of sustainable funding for the use of bivalent vaccines, and market incentives to ensure vaccine supply.

## Conclusions

Despite the advances in the control of enteric fever caused by *Salmonella* Typhi worldwide, the development of a vaccine that addresses enteric fever more broadly by adding protection against Salmonella Paratyphi A remains a priority for endemic countries. Moreover, the WHO’s vision calls for a broadly protective *Salmonella* vaccine for the prevention of invasive disease caused by both typhoidal and non-typhoidal *Salmonella* (NTS) serovars. Quadrivalent vaccines covering the four predominant *Salmonella* serovars: *Salmonella* Typhi, Paratyphi A, Typhimurium, and Enteritidis, are increasingly recognized as a strategic priority, particularly in the context of minimizing childhood injections. ^62^ The bivalent *Salmonella* Typhi/Paratyphi A vaccine represents a first step towards that vision and, in conjunction with national surveillance and laboratory capacity strengthening, and ongoing improvements in WASH practices, has the potential to significantly reduce the burden of infection and mitigate the spread of AMR in low-and-middle income countries, where populations are most impacted.
